# Potential application of the haematology analyser XN-31 prototype for field malaria surveillance in Kenya

**DOI:** 10.1186/s12936-022-04259-7

**Published:** 2022-09-01

**Authors:** Wataru Kagaya, Ikki Takehara, Kyoko Kurihara, Michael Maina, Chim W. Chan, Gordon Okomo, James Kongere, Jesse Gitaka, Akira Kaneko

**Affiliations:** 1 Department of Virology and Parasitology/Research Center for Infectious Disease Sciences, Graduate School of Medicine, Osaka Metropolitan University, 1-4-3, Asahimachi, Abeno-ku, Osaka, 545-8585 Japan; 2 Sysmex Corporation, 4-4-4 Takatsukadai, Nishiku, Kobe, Hyogo 651-2271 Japan; 3 Department of Clinical Medicine, Mount Kenya University, PO Box 342-01000, Thika, Kenya; 4 Homa Bay County Government, Homa Bay, Kenya; 5 Nairobi Research Station, Nagasaki University Institute of Tropical Medicine-Kenya Medical Research Institute (NUITM-KEMRI) Project, Institute of Tropical Medicine (NEKKEN), Nagasaki University, PO Box 19993-00202, Nairobi, Kenya; 6 Institute of Tropical Medicine (NEKKEN), Nagasaki University, 1-12-4 Sakamoto, NagasakiNagasaki, 852-8523 Japan; 7 Centre for Malaria Elimination, Mount Kenya University, P.O. Box 342-01000, Thika, Kenya; 8 Island Malaria Group, Department of Microbiology, Tumor and Cell Biology (MTC), Karolinska Institutet, Biomedicum, Solnavägen 9, Solna, 171 65 Stockholm, Sweden; 9 Department of Virology and Parasitology, Graduate School of Medicine, Osaka Metropolitan University, 1-4-3, Asahimachi, Abeno-ku, Osaka, 545-8585 Japan

## Abstract

**Background:**

Simple and accurate diagnosis is a key component of malaria control programmes. Microscopy is the current gold standard, however it requires extensive training and the results largely rely on the skill of the microscopists. Malaria rapid diagnostic tests (RDT) can be performed with minimal training and offer timely diagnosis, but results are not quantitative. Moreover, some *Plasmodium falciparum* parasites have evolved and can no longer be detected by existing RDT. Developed by the Sysmex Corporation, the XN-31 prototype (XN-31p) is an automated haematology analyser capable of detecting *Plasmodium*-infected erythrocytes and providing species differentiation and stage specific parasite counts in venous blood samples without any preparation in approximately one minute. However, factors such as stable electricity supply in a temperature-controlled room, cost of the instrument and its initial set-up, and need for proprietary reagents limit the utility of the XN-31p across rural settings. To overcome some of these limitations, a hub and spoke diagnosis model was designed, in which peripheral health facilities were linked to a central hospital where detection of *Plasmodium* infections by the XN-31p would take place. To explore the feasibility of this concept, the applicability of capillary blood samples with the XN-31p was evaluated with respect to the effect of sample storage time and temperature on the stability of results.

**Methods:**

Paired capillary and venous blood samples were collected from 169 malaria-suspected outpatients in Homa Bay County Referral Hospital, Kenya. Malaria infections were diagnosed with the XN-31p, microscopy, RDT, and PCR. Capillary blood samples were remeasured on the XN-31p after 24 h of storage at either room (15–25 °C) or chilled temperatures (2–8 °C).

**Results:**

Identical results in malaria diagnosis were observed between venous and capillary blood samples processed immediately after collection with the XN-31p. Relative to PCR, the sensitivity and specificity of the XN-31p with capillary blood samples were 0.857 and 1.000, respectively. Short-term storage of capillary blood samples at chilled temperatures had no adverse impact on parasitaemia and complete blood counts (CBC) measured by the XN-31p.

**Conclusion:**

These results demonstrate the potential of the XN-31p to improve routine malaria diagnosis across remote settings using a hub and spoke model.

**Supplementary Information:**

The online version contains supplementary material available at 10.1186/s12936-022-04259-7.

## Background

Even though malaria is one of the oldest infectious diseases, it remains a major public health problem worldwide. In 2020, there was an estimated 241 million malaria cases and 627,000 malaria deaths globally [1]. After expanding the usage of novel tools such as rapid diagnostic test (RDT), insecticide treated bed net (ITN), and artemisinin-based combination therapy (ACT), these epidemiological indices have decreased remarkably since 2000. However, the reduction has slowed recently, with the number of cases slightly increasing in the last five years and the uptick further exacerbated by the COVID-19 pandemic [1]. These trends suggest a need for developing more effective diagnostics and treatments, improved and innovative vector control methods, and other tools such as vaccines [2].

In malaria diagnosis, microscopic examination of Giemsa-stained blood films relies on the visual detection of parasites, allows for estimate of parasite density, and remains the gold standard. However, training for competent microscopists takes time [3]. Also in elimination settings, malaria microscopy becomes more challenging because decreasing malaria prevalence presents fewer opportunities for microscopists to observe parasites and to maintain competence [4], and infections are more likely to have parasite density below the detection limits [5, 6]. On the contrary, RDT requires less training, is more convenient, and can give results within fifteen minutes. Thus in many remote settings, RDT has effectively replaced microscopy for routine diagnosis at health facilities as well as active case detection by community health workers. However, since RDT is not quantitative and does not differentiate the various parasite developmental stages, it has limited utility in determining the effect of treatment or monitoring the impact on transmission [7]. Furthermore, recent reports of RDT false negativity due to deletions of the gene encoding the target histidine rich protein 2/3 (HRP2/3) in *Plasmodium falciparum* have raised questions about diagnostic accuracy [8–10]. *Plasmodium* lactate dehydrogenase (pLDH)-detecting RDTs are also available, but they have lower sensitivity compared with HRP2-detecting RDT [11]. Polymerase chain reaction (PCR) has higher sensitivity and specificity relative to microscopy and RDT and is widely used for research purposes, but is not practical for clinical use since it requires more advanced equipment and skills and is more expensive. To complement existing methods, there exists a need for innovations in malaria diagnosis, especially those that are highly sensitive and easy to operate in malaria endemic settings.

The automated haematology analyser XN-31 prototype (XN-31p) (Sysmex Corporation, Kobe, Japan) is a novel malaria diagnostic support system developed for the clinical setting. The analyser is based on the principle of fluorescence flow cytometry and shares the same physical characteristics, hardware configuration, and reagent requirements with the XN-30 that is used extensively for research. Both analysers can measure a complete blood count (CBC), count malaria-infected red blood cells (iRBC), and calculate parasitaemia with a limit of quantification of 20 iRBC/μL in approximately 1 minute ^12^. These analysers offer three measurement modes with different sample volumes and different limit of quantification. Previous studies from different malaria endemic settings showed that they had comparable performance to microscopy and RDT in malaria diagnosis and good concordance with microscopy and quantitative PCR (qPCR) in parasite counts [12–14]. However, venous blood samples were used in all previous evaluations except one [14], whereas for routine malaria diagnosis in health facilities capillary blood obtained by finger prick is used. More evidence for the applicability of capillary blood for malaria diagnosis by the XN-31p is needed.

Currently the operation of the XN-31p requires a stable electricity supply in a temperature-controlled room and specialised reagents not widely available in many remote settings. The cost of the instrument also precludes widespread deployment in developing countries. To address some of these limitations, a hub and spoke diagnosis model was designed, whereby blood samples collected in peripheral health facilities would be transported to a central hospital for malaria diagnosis by the XN-31p. The effects of transport time and temperature on sample integrity in such model were evaluated by comparing the results from capillary blood samples measured immediately after sample collection with those measured 24 h after storage at either room or chilled temperatures. Results from this study provided support for the use of the XN-31p in a hub and spoke model to cover a wider range of settings for routine malaria diagnosis.

## Methods

### Study design and study site

An observational cross-sectional study was conducted to compare the effects of blood sampling methods (venous vs. capillary) and sample storage temperature and length on the performance of the XN-31p in the detection of *Plasmodium* infections. The study was conducted in Homa Bay County Referral Hospital in Kenya between March and April 2020. The hospital is the major referral hospital in Homa Bay County, accepting patients throughout the county. As a normal clinical procedure, all outpatients were first examined by clinical officers. Once malaria was suspected, the patient was included in the study after obtaining informed consent.

Homa Bay County lies along the eastern shore of Lake Victoria and is among the regions with the highest malaria prevalence in Kenya [15]. Two rainy seasons are observed in the Lake Victoria basin: the long rainy season runs from March to June and the short rainy season from November to December. Malaria incidence peaks one to two months after the rainy seasons. The average *P. falciparum* parasite prevalence in 2–10 years old in the Lake Victoria region in Kenya is estimated to be more than 30% [15]. Long-lasting insecticidal nets (LLINs) were distributed for free by the Ministry of Health in 2017, but the proportion of households with at least one LLIN for every two persons was only 50% in the Lake region [16]. IRS has been conducted across Homa Bay County once in a year since 2018 [17], and the third round of IRS was implemented in February and March 2020, just prior to this study [18]. A pilot introduction of the RTS,S/AS01 vaccine was started in September 2019 [19].

### Procedures for the sample collection

Prior to blood sampling, a questionnaire was administered to study participants to obtain sociodemographic information and known malaria risk factors. Capillary blood (250 µL) was obtained by finger prick in a BD K2-EDTA Microtainer blood collection tube (BD, New Jersey, US). After inversions, 70 µL of blood was spotted on Whatman ET31 Chr filter paper (Whatman International, Maidstone, UK) and stored in a zipped plastic bag after drying at room temperature. Capillary blood was also used to prepare thick and thin blood smears for microscopic examination, and for RDT diagnosis using the SD Bioline Malaria Ag Pf/Pv RDT (Standard Diagnostics Inc., Gyeonggi-do, South Korea). A detailed protocol of capillary blood collection was shared with laboratory technicians (Additional file 1: Fig. S2). Venous blood (500 µL) was collected with a hypodermic needle and syringe and transferred to a different BD K2-EDTA Microtainer blood collection tube.

The R-based statistical software EZR [20] was used to estimate the sample size needed for a non-inferiority test for two proportions, with the expected sensitivity of XN-31p and the sensitivity of microscopy at 0.95, a margin of non-inferiority at 0.05, significance level at 0.05, and power at 0.8. The minimum sample size was 235.

### Diagnostic methods: measurement with XN-31p, microscopy, RDT, and PCR

All diagnostic methods except PCR were carried out at the Homa Bay County Referral Hospital laboratory. PCR was performed in Osaka City University, Japan.

Immediately after sampling, both capillary and venous blood samples were directly analysed on the XN-31p (Sysmex Corporation, Kobe, Japan) using the low malaria (LM) mode [21]. In LM mode, approximately 130 µL of blood is needed, although only 60 µL of the sample is used for analysis. Detailed features of the XN-31p were described in a previous report [14].

Based on the results of this initial analysis, samples were divided into two groups and stored at either room or chilled temperature for 24 h. Samples in the room temperature group (8 positive and 39 negative samples) were kept in an air-conditioned room with temperature set at 22 °C, while those in the chilled temperature group (8 positive and 43 negative samples) were kept in a cooler box with ice packs. The ice packs were changed in the morning. Temperatures taken several times over a 24-h period indicated that the room was between 15 and 25 °C and the cooler box between 2 and 8 °C. After 24 h all samples were analysed again on the XN-31p using the LM mode.

Microscopic examination was performed as described in the malaria microscopy standard operating procedure (MM-SOP-09) by the World Health Organization (WHO) [22]. Briefly, thin smears were fixed with methanol, and both thin and thick smears were stained with 3% Giemsa solution for 30 min. Samples were deemed positive if parasites were observed in the thick smear. %iRBC and iRBC counts/µL were calculated as described in Additional file 1: Fig. S3. The slides were examined by two experienced microscopists and samples with discordant results were further examined by a third experienced microscopist who was blind to results from the first two readings. In addition to *Plasmodium* parasites, the presence of sickle cell, codocyte, dacrocyte, Howell-Jolly body (if present, the count), haemolysis, morphological abnormality of WBC, fibrin clots, and platelet aggregation was noted. RDT results were obtained following manufacturer’s instructions. For PCR diagnosis, DNA was extracted from a quartered blood spot (17.5 µL) using the QIAamp Blood Mini Kit (Qiagen, Hilden, Germany) according to the manufacturer’s instructions. PCR amplification of the *Plasmodium* mitochondrial cytochrome c oxidase III (*cox3*) gene was performed as described previously [23].

### Statistical analyses

All questionnaire data together with RDT, microscopy, and PCR results were entered into a Microsoft Excel spreadsheet and cross-checked for errors. The sensitivity, specificity, positive and negative predictive values of the XN-31p were calculated in reference to RDT, microscopy, and PCR using the R-based statistical software EZR [20]. Concordance in malaria parasitaemia and CBC between capillary and venous blood samples, between the XN-31p and microscopy, and between the initial measurement and that after 24 h at room temperature or cool condition was evaluated by the Bland–Altman plot method using GraphPad Prism 9.2.0 (GraphPad Software Inc, CA, USA). Wilcoxon matched pair signed rank test, and Spearman’s rank correlation test were performed to examine the presence of proportional bias and fixed bias, respectively with STATA/MP 16.1 (StataCorp, TX, USA).

## Results

### Participant characteristics

Of 171 subjects enrolled in this study, 169 provided both venous and capillary blood samples for evaluation of the XN-31p. The demographic and clinical characteristics of the participants are described in Table 1. The number of participants with fever at enrolment was 33 (19.8%). The median white blood cell (WBC) count was 5.93 × 10^3^/μL, and the prevalence of anaemia, defined as haemoglobin (Hb) of < 11 g/dL, was 20.1%.

**Table 1:** Participant demographic and clinical characteristics at enrolment

	Total enrolled (n = 169)
Age; median (IQR)	23 (5–36)
Male; n (%)	75 (44)
Febrile subjects (≧37.5); n (%)	33 (19.8)
Duration of fever (days); median (IQR)	3 (2–3)
Having chronic conditions; n (%)	13 (7.7)
Took antimalarials in the last one month; n (%)	13 (7.7)
Travelled in the last 3 months; n (%)	13 (7.7)
Slept under a bed net in previous night; n (%)	155 (91.7)
Covered by IRS programme for the last one year; n (%)	119 (70.4)
Enrolled in RTS,S vaccine trial; n (%)	3 (1.9)
Pregnancy; n (% of total female)	4 (2.4)
Haematological parameters*	
White blood cell (10^3^/μL); median (IQR)	5.93 (4.82–8.93)
Red blood cell (10^6^/μL); median (IQR)	5.04 (4.67–5.46)
Haemoglobin (g/dL); median (IQR)	13.1 (11.5–14.2)
Anaemia (Hb < 11 g/dL); n (%)	34 (20.1)
Haematocrit (%); median (IQR)	39.8 (34.6–43.1)
Platelets (10^3^/μL); median (IQR)	256 (197–364)
Thrombocytopenia (< 150 × 10^3^/μL); n (%)	14 (8.3)

^*^Measured with venous blood in LM mode on the XN-31p

### Diagnostic performance of XN-31p

Table 2 shows the number of malaria cases detected by each method. The XN-31p, microscopy, RDT, and PCR detected 18, 16, 18, and 23 *Plasmodium* infections, respectively. One *Plasmodium malariae* infection was detected by microscopy while three *Plasmodium ovale* and one mixed infection of *P. falciparum* and *P. malariae* were detected by PCR. On the XN-31p, *Plasmodium* infections were detected in the same set of 18 samples using both capillary and venous blood; all were identified as *P. falciparum* except in one capillary blood sample where the species could not be specified. Inconclusive results (MI-RBC abnormal [Abn] Scattergram) were reported in five venous and eleven capillary blood samples by the XN-31p. Representative examples of normal and abnormal scattergrams are shown in Additional file 1: Fig. S1. Of the five venous blood samples with MI-RBC Abn Scattergram, four showed the same inconclusive result with their corresponding capillary blood samples. The median parasite densities determined by the XN-31p using venous blood, capillary blood, and microscopy were 21,774, 21,357, and 2,356 parasites/μL, respectively.

**Table 2:** Diagnosis of malaria by different methods

	XN-31p (venous blood)	XN-31p (capillary blood)	Microscopy	RDT	PCR
*Plasmodium* positive; n (%)	18 (10.7)	18 (10.7)	16 (9.47)	18 (10.7)	23 (13.6)
* P. falciparum*	18	17	15	18	19
Others*	0	1	–	–	–
* P. malariae*	–	–	1	–	0
* P. ovale*	–	–	0	–	3
* P. falciparum* + *P. malariae*	–	–	0	–	1
MI-RBC Abn Scattergram	5	11	–	–	–
Negative	146	140	153	151	146
Parasitaemia (parasite/μL); median (IQR)	21,774 (9,490 −101,973)	21,357 (9,262 −104,193)	2,356 (1,004 −18,910)	–	–
Parasitaemia (%iRBC); median (IQR)	0.44 (0.20–1.8)	0.45 (0.19–1.8)	0.27 (0.15–0.92)	–	–

^*^Except *P. falciparum*

Comparisons of the performance of the XN-31p with other diagnostic methods are described in Table 3. Samples showing MI-RBC Abn Scattergram were excluded in these comparisons. Performance measures of the XN-31p against microscopy, RDT, and PCR were similar, with high sensitivity (0.818–1.000), specificity (0.986–1.000), positive predictive value (PPV; 0.889–1.000) and negative predictive value (NPV; 0.973–1.000). Against microscopy and RDT, all performance measures of the XN-31p were identical between capillary and venous blood. Against PCR, slight differences in sensitivity and NPV were found between capillary and venous blood. Detailed comparisons including samples with MI-RBC Abn Scattergram are shown in Additional file 1: Table S1.

**Table 3:** Assessment of the diagnostic performance of the XN-31p using microscopy, RDT, and PCR as standards

	Microscopy		RDT		PCR	
	Pos	Neg	Pos	Neg	Pos	Neg
XN-31p (venous blood)						
Pos	16	2	18	0	18	0
Neg	0	146	0	146	4	142
	Value	95% CI	Value	95% CI	Value	95% CI
Sen	1.000	0.713–1.000	1.000	0.740–1.000	0.818	0.597–0.948
Spe	0.986	0.952–0.998	1.000	0.963–1.000	1.000	0.962–1.000
PPV	0.889	0.653–0.986	1.000	0.740–1.000	1.000	0.740–1.000
NPV	1.000	0.963–1.000	1.000	0.963–1.000	0.973	0.931–0.992
XN-31p (capillary blood)						
Pos	16	2	18	0	18	0
Neg	0	140	0	140	3	137
	Value	95% CI	Value	95% CI	Value	95% CI
Sen	1.000	0.713–1.000	1.000	0.740–1.000	0.857	0.637–0.970
Spe	0.986	0.950–0.998	1.000	0.961–1.000	1.000	0.960–1.000
PPV	0.889	0.653–0.986	1.000	0.740–1.000	1.000	0.740–1.000
NPV	1.000	0.961–1.000	1.000	0.961–1.000	0.979	0.939–0.996

*Pos* positive, *Neg* negative, *Sen* sensitivity, *Spe* Specificity, *PPV* positive predictive value, *NPV* negative predictive value

### Concordance in parasitaemia and CBC between capillary blood and venous blood

**Fig. 1:**
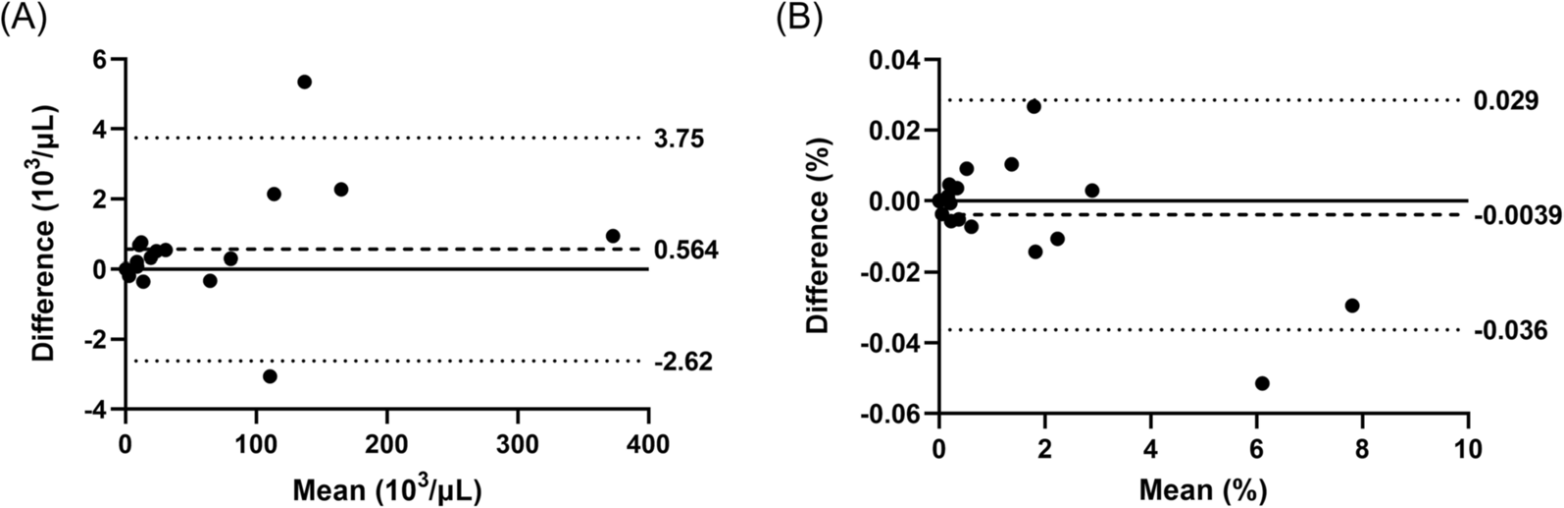
Bland–Altman analyses of concordance in **A** iRBC count per µL and **B** %iRBC between venous and capillary blood samples measured by XN-31p

The concordance in parasitaemia between capillary and venous blood was evaluated by Bland–Altman analysis (Fig. 1). The limits of agreement were between −2.622 and 3.750 × 10^3^ for iRBC count per μL, and between −0.036 and 0.029% for %iRBC. There were both fixed and proportional biases in the iRBC count per μL (p < 0.05 with Wilcoxon matched pairs signed rank test, rho = 0.515, p < 0.05 with Spearman's rank correlation test), but not in iRBC%.

**Fig. 2:**
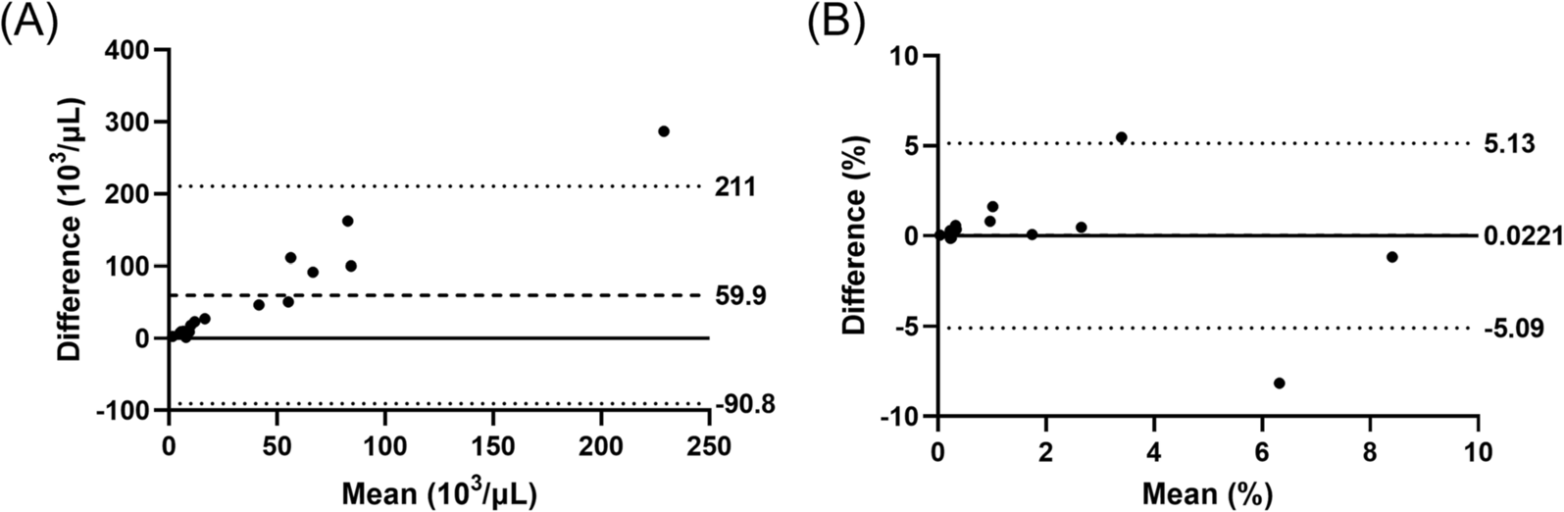
Bland–Altman analyses of concordance in **A** iRBC count per µL and **B** %iRBC in capillary blood between microscopy and XN-31p

For capillary blood samples, parasitaemia determined by microscopy and XN-31p showed significant correlation in both iRBC count per μL (rho = 0.538, p < 0.05), and %iRBC (rho = 0.643, p < 0.05). For iRBC count per μL, Bland–Altman analysis showed poor concordance between microscopy and XN-31p, with the limits of agreement between -90 and 210 × 10^3^ iRBC/μL and fixed and proportional biases (p < 0.001 with Wilcoxon matched-pairs signed rank test, rho = 0.9412, p < 0.001 with Spearman's rank correlation test) (Fig. 2a). On the other hand, for %iRBC microscopy and XN-31p showed good concordance without fixed and proportional bias. The limit of agreement was between -5.09 and 5.13%iRBC (Fig. 2b).

**Fig. 3:**
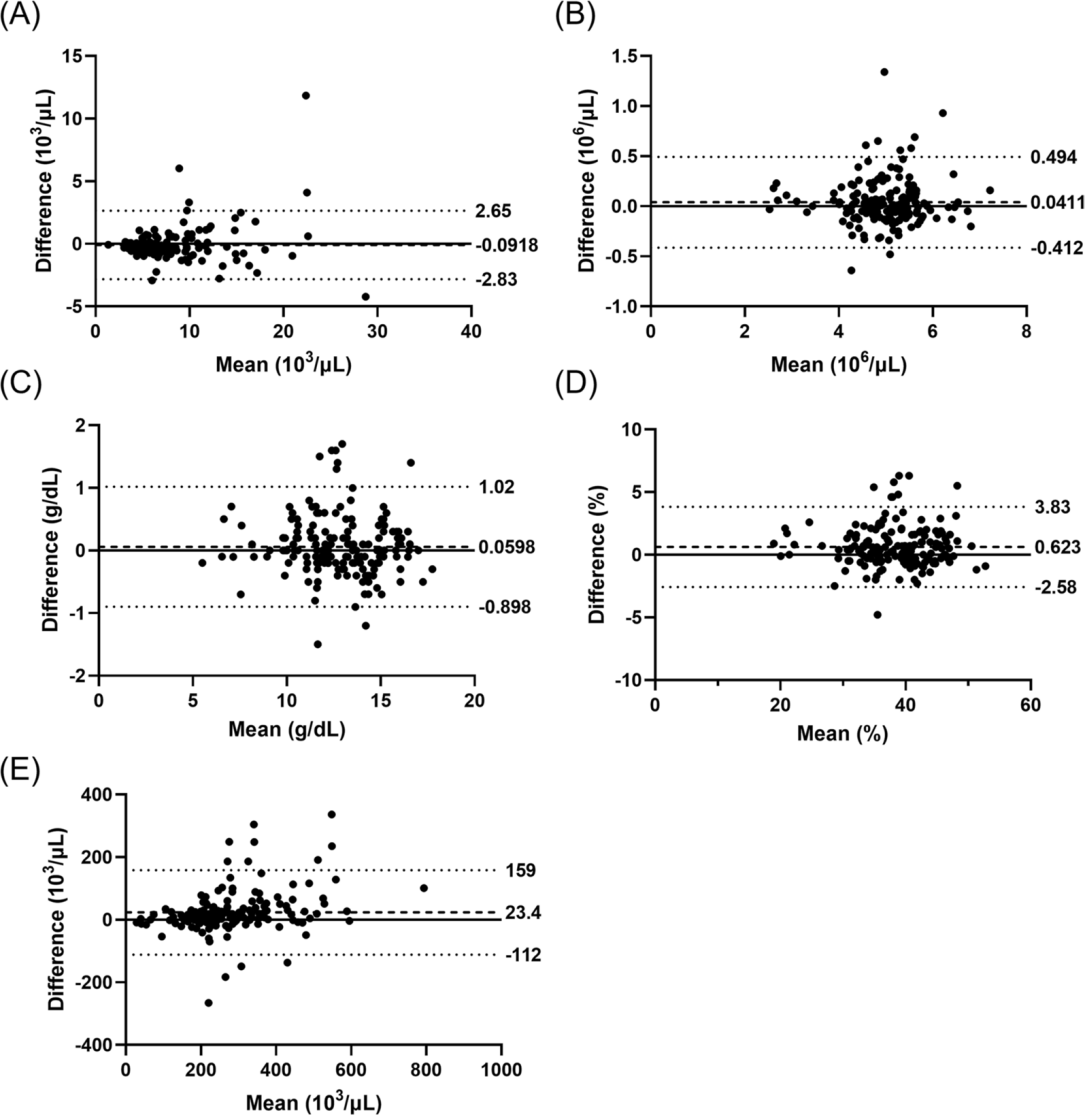
Bland–Altman analyses of concordance in **A** WBC, **B** RBC, **C** Hb, **D** haematocrit, and **E** platelet between venous and capillary blood samples measured by XN-31p

Bland–Altman analyses of the concordance in CBCs between venous and capillary blood samples are shown in Fig. 3. Fixed biases (all p < 0.001) were observed in WBC, haematocrit (HCT) and platelet while proportional bias was found only in platelet (rho = 0.344, p < 0.001).

### Stability of iRBC and CBCs by time and temperature

**Fig. 4:**
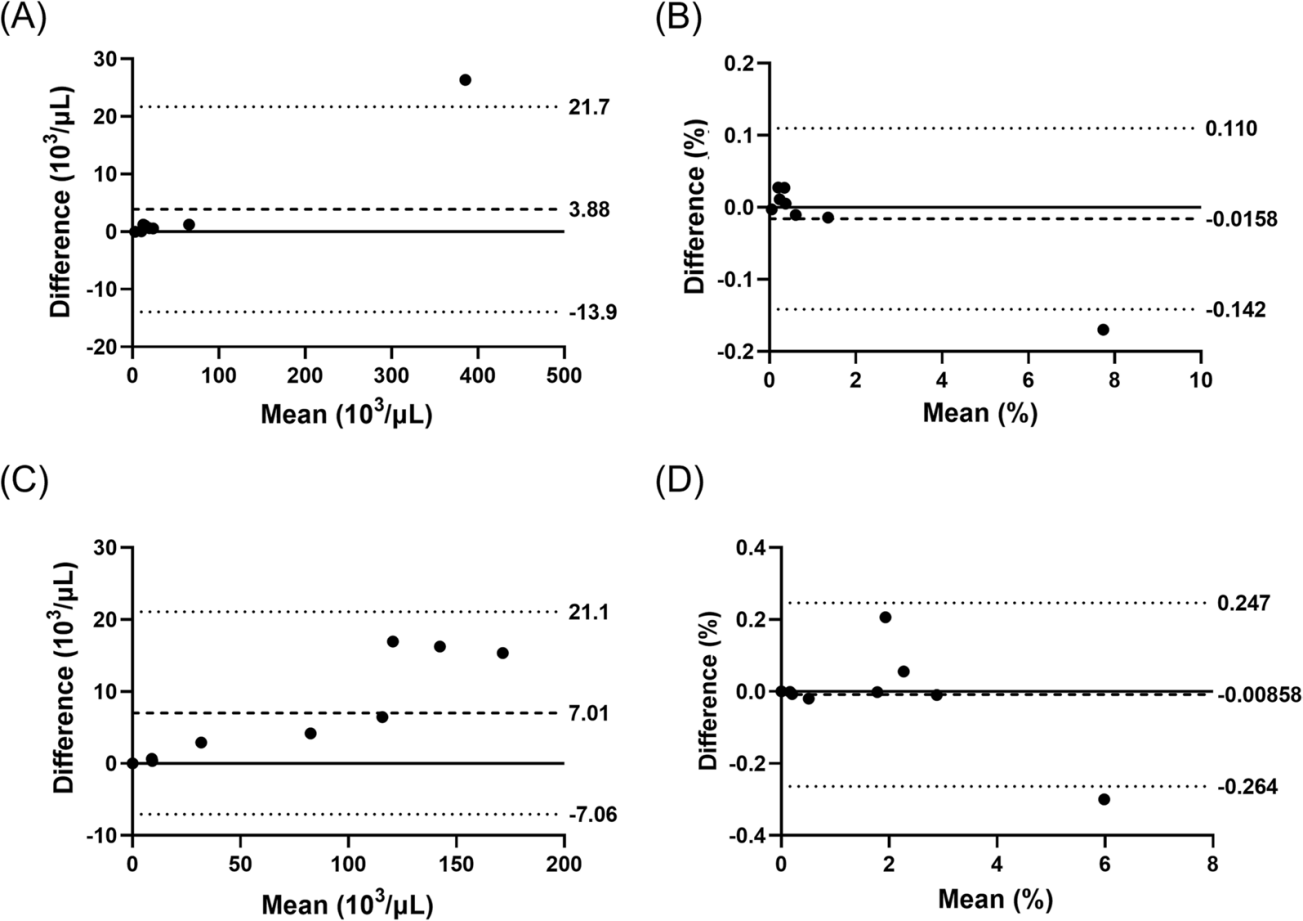
Effects of storage time and temperature on parasitaemia in capillary blood samples measured by XN-31p. Bland–Altman analyses of concordance in **A** iRBC count per µL after 24 h at 2–8 °C, **B** %iRBC after 24 h at 2–8 °C, **C** iRBC count per µL after 24 h at room temperature, and **D** %iRBC after 24 h at room temperature

Capillary blood samples were processed with the XN-31p again after 24 h at either room (15–25 °C) or chilled temperature (2–8 °C). Bland–Altman analyses indicated good concordance in both iRBC count per μL and %iRBC after 24 h, although results were more consistent for samples stored at chilled temperatures and samples with lower parasitaemia (Fig. 4).

**Fig. 5:**
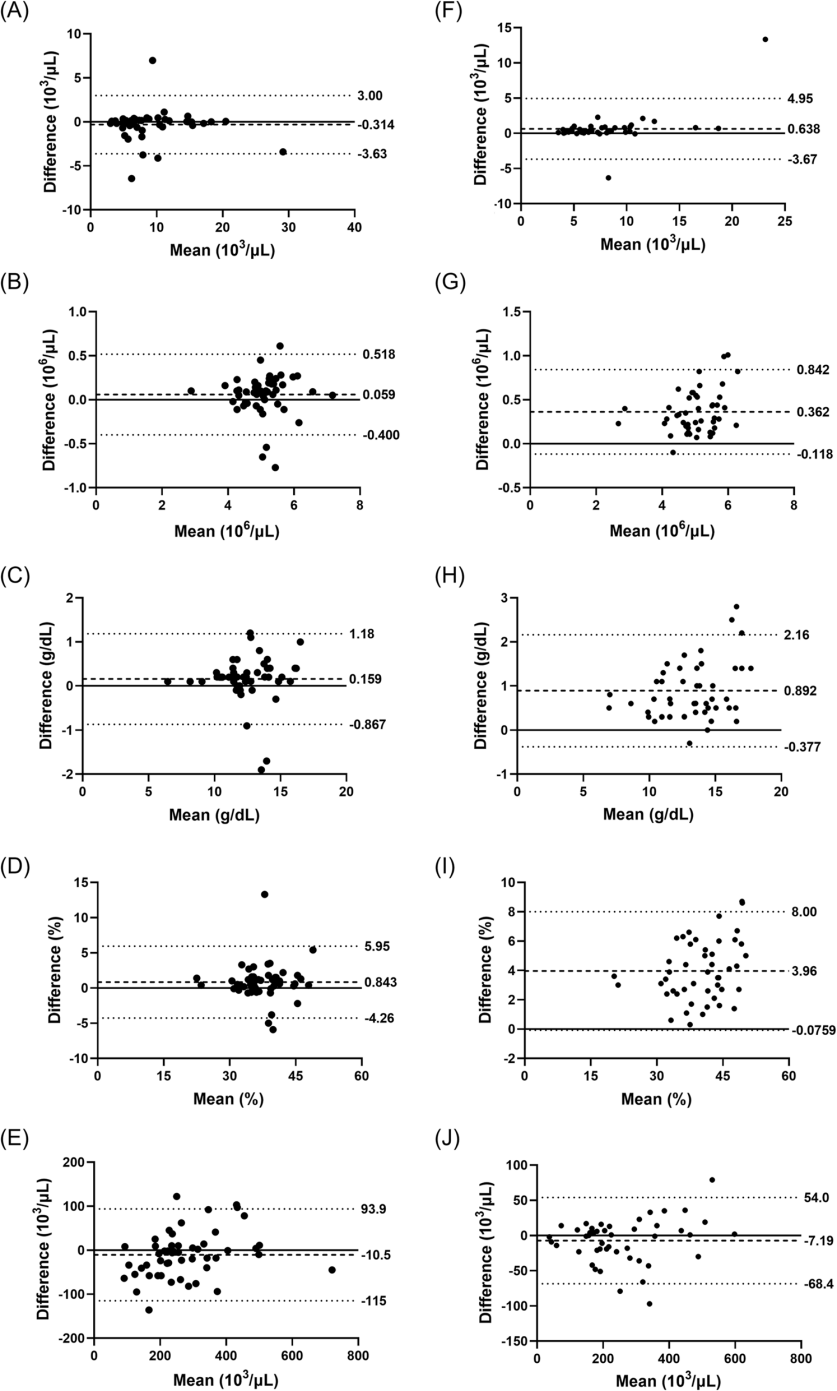
Effect of storage temperature on concordance in the complete blood counts (CBCs) measured by XN-31p. Capillary blood samples were kept for 24 h at either chilled (2 to 8 °C; **A** through **E**) or room temperature (15 to 25 °C; **F** through **J**). WBC (**A** and **F**), RBC (**B** and **G**), Hb (**C** and **H**), haematocrit (**D** and **I**), and platelet (**E** and **J**) were measured

The stability of CBCs is shown in Fig. 5. Samples stored for 24 h at 2–8 °C had better concordance with the original measurements at 0 h than those stored at room temperature. The biases detected in each measurement are summarised in Additional file 1: Table S2.

## Discussion

Early diagnosis and treatment are key to malaria control. Currently malaria diagnosis in the field largely relies on microscopy and RDT, however both methods have limitations. Furthermore, malaria parasites are evolving to evade diagnosis, as demonstrated by the deletions of the *pfhrp2* and *pfhrp3* genes that allow parasites to escape detection by RDT [24]. Thus innovative tools are needed to overcome existing challenges toward global malaria elimination. In this study, a novel malaria diagnostic system, the Sysmex XN-31p was compared with conventional diagnostic methods in the Lake Victoria region, Kenya, and its applicability for use in the field was evaluated. Several studies have shown the capability of the XN-31p and its predecessor, the XN-30, to diagnose *Plasmodium* infections in different settings [12–14], but this is the first report showing the concordance of results between venous and capillary blood samples without any pre-treatment or sample dilution prior to analysis. Also this study shows that parasitaemia results obtained from capillary blood were stable even after 24 h, especially if the samples can be kept at chilled conditions. These positive findings unlock the potential use of the XN-31p as a diagnostic system for both clinical use and mass blood surveys in remote settings.

The diagnostic performance of XN-31p is quite comparable to that of existing field diagnostics, namely microscopy and RDT. The XN-31p was highly sensitive (1.000) and specific (0.986–1.000) against these conventional methods. These results are in good agreement with previous reports [12–14]. More importantly the performance was reproducible using capillary blood samples without any pre-treatment or dilution.

Detection of low-density *Plasmodium* infections by PCR is common in research but remains limited in clinical settings. Relative to PCR, the XN-31p detected fewer *Plasmodium* infections, resulting in a lower sensitivity (0.818–0.857) but maintaining a very high specificity (1.000). The lower diagnostic performance of the XN-31p is a disadvantage, however when compared to PCR, the XN-31p is easier to operate and provides results in a shorter period of time, making the XN-31p more suitable in clinical settings.

The XN-31p has three measurement modes. Whole blood (WB) and LM modes do not require any pre-treatment prior to the measurement, while PD mode needs sample dilution before the measurement. PD mode requires only 20 μL of blood and thus is useful when the amount of available sample is very limited. The applicability of capillary blood in PD mode was previously tested, however our own preliminary tests indicated low consistency in parasite counts and CBCs due to errors introduced during sample dilution. LM mode uses 130 μL of blood, which can be readily obtained through finger prick. Our comparison showed excellent concordance between venous and capillary blood samples for malaria diagnosis and species differentiation when samples were measured in LM mode. Good concordance was also observed for %iRBC, with the limits of agreement between −0.036 and 0.029%, which are well accepted in the clinical context. However, the parasite count per μL had fixed and proportional biases and the ranges for the limits of agreement were quite wide. Since there was no significant difference in RBC counts between capillary and venous blood samples (as shown in Fig. 3), the discrepancy must have derived from actual differences in iRBC count. The cause of differences in iRBC count is under investigation.

There was a high correlation but low concordance between the parasitaemia determined by the XN-31p and microscopy. By microscopy, parasite density was determined by counting the number of iRBC against 200 WBCs in the thick blood smear and assuming 8,000 WBCs per μL of blood. In this study, WBC counts were quite variable among participants (Fig. 3A), which might have contributed to the discordance. However, %iRBC was obtained without any assumption, thus the gap observed here may be purely due to the difference of two measurements. Similar trends in the concordance of parasitaemia between the XN-31p and microscopy were reported previously [14]. Although microscopy is considered the gold standard in malaria diagnosis, accurate determination of parasitaemia depends on a number of factors including the skills of microscopists and the quality of the stained blood smears, thus other quantitative methods such as qPCR may give insights into the accuracy of parasite count with XN-31p.

Malaria-positive samples are flagged by the XN-31p as “Malaria?(P.f.)”, “Malaria?(Others)” or “Malaria?(UNC)” based on the pattern of scattergram. Unfortunately, few non-*P. falciparum* malaria infections were included in this study, making it difficult to evaluate the ability of XN-31p to differentiate *Plasmodium* species. All *P. ovale* infections in this study were detected by PCR only and thus were likely to have low parasitaemia. It is reported that *P. ovale* infections tend to have lower parasitaemia because of the species preference to invade reticulocytes [25]. PCR detected one case of *P. falciparum*-*P. malariae* co-infection, which was not detected by other methods likely due to low parasitaemia. It would be interesting to test the diagnostic capability of the XN-31p in mixed-species infections since the scattergram patterns of RBC infected by different *Plasmodium* species may overlap with one another. No *Plasmodium vivax* was observed in this study, consistent with a previous report from the same area [26].

A small number of samples in our study had inconclusive results, reported as MI-RBC Abn Scattergram by the XN-31p. In the case of capillary blood, a previous study attributed this scattergram to the presence of Howell-Jolly body or crystalized Sickle haemoglobin (HbS) [12]. However in this study, HbS was reported in only 3 of 11 samples with MI-RBC Abn Scattergram, and the one sample reporting the presence of Howell-Jolly body did not report MI-RBC Abn Scattergram. Preliminary tests revealed a relatively high number of results reporting MI-RBC Abn Scattergram, which were likely caused by the finger prick method to obtain capillary blood. Repeated application of pressure on the finger can cause mechanical damage or deformation to blood cells, and damaged cells form aggregates with platelet and fibrin. The observation that inconclusive results were reported in more than twice as many capillary blood (11) as venous blood (5) samples is consistent with this hypothesis. To reduce the appearance of MI-RBC Abn Scattergram in this study, all laboratory technicians were retrained to follow the blood sampling procedure recommended by the Clinical and Laboratory Standards Institute (CLSI) (Additional file 1: Fig. S2). Interestingly most of the MI-RBC Abn Scattergram (80% for venous blood samples and 81.8% for capillary blood samples) were observed in PCR negative samples in this study. Further tests with a larger sample size may provide a clearer answer to the causes of MI-RBC Abn Scattergram.

Operations of the XN-31p require stable electricity supply that are often unavailable in health facilities providing services in malaria endemic areas. To extend the utility of XN-31p as a malaria diagnostic tool in remote communities, capillary blood samples kept at either room or chilled temperatures after 24 h were remeasured to simulate the time and condition of blood sample transport from distant villages to Homa Bay County Referral Hospital. Generally, samples stored at 2–8 °C had better concordance in parasitaemia with their 0-h results than samples stored at room temperature, with narrower limits of agreement. The concordance would be higher if one outlier were excluded, though the numbers of *Plasmodium* positive samples available for analyses were small. CBC data also showed better concordance in samples stored at chilled temperatures. Samples were kept cold in an inexpensive, food-grade insulated cooler box with ice packs that can be readily maintained in basic health facilities. Some studies showed the correlation between the distance from health facility and malaria prevalence [27–30]. The hub and spoke model described in this study can be exploited to advance universal access to malaria diagnosis and treatment.

One of the unique features of the XN-31p is the simultaneous detection of *Plasmodium* infection and measurement of CBCs, which may be useful in malaria eliminating areas. In such settings, lower malaria prevalence means fewer chances for microscopists to examine blood films and maintain competence in malaria diagnosis. Moreover, differential diagnosis of febrile diseases becomes more important in eliminating settings where malaria is no longer the main cause of fever [31–34]. The XN-31p can improve fever case management by excluding malaria as the cause of febrile illnesses. Combined with differential WBC counts, the XN-31p can further help clinical practitioners in rural health facilities to prescribe appropriate treatments.

This is the first test of the XN-31p in East Africa. The Lake Victoria basin has some of the highest malaria burden in Kenya, and previous cross-sectional surveys in Homa Bay County showed malaria prevalence of approximately 20% and 40% by microscopy and PCR, respectively [26, 35]. Malaria prevalence in the study area has decreased substantially due partly to the roll out of the indoor residual spraying (IRS) programme. Also the onset of the COVID-19 pandemic coincided with the start of this study, greatly reducing the number of outpatients seeking treatment at the hospital and resulting in a study sample size much smaller than anticipated due to altered care-seeking behaviours driven by social distancing measures such as lock downs and travel restrictions. In this study site, almost all outpatients presenting fever and/or malaria-related symptoms at health facilities are routinely tested for malaria. Thus many non-malarial cases were recruited for this study; only about 10% of the study participants were malaria positive. However, the results from this study are in general agreement with those from previous reports and provide evidence to support further development and evaluation of the XN-31p as a malaria diagnostic tool.

In summary, this study showed that in lieu of venous blood, capillary blood can be used directly without any pre-treatment or dilution for malaria diagnosis on the XN-31p automated haematology analyser. Moreover, capillary blood can be stored at chilled temperature for up to 24 h without adversely affecting malaria diagnostic and CBC results, broadening the appeal to utilise the XN-31p in a hub and spoke model as a rapid and accurate malaria diagnostic method for mass surveys and case confirmation in remote locations. The ability of the XN-31p to detect asymptomatic and submicroscopic infections and mixed-species infections needs to be investigated.

## Supplementary Information

**Additional file 1: Fig. S1.** Scattergrams illustrating (a) malaria-negative sample, (b) *P. falciparum* positive sample, and (c) inconclusive (MI-RBC abnormal [Abn] scattergram) by XN-31p. SFL: side fluorescence light; FSC: forward scattered light. Blue dots: non-infected RBCs, platelets, and debris; red dots: parasite-infected red blood cells; light blue dots: white blood cells. **Fig. S2.** Protocol for capillary blood collection. **Fig. S3.** Flow chart of iRBC count and %iRBC determination by microscopy. **Table S1.** Performance of XN-31p in comparison to conventional methods with detailed results. **Table S2.** Fixed and proportional biases observed in the CBCs with 24 hours stored samples

## Data Availability

The datasets used and/or analysed during the current study are available from the corresponding author on reasonable request.
