# Supplementary material for: Potential application of the haematology analyser XN-31 prototype for field malaria surveillance in Kenya
Source: Malar J. 2022 Sep 1;21:252. doi: 10.1186/s12936-022-04259-7 (PMC9434510; doi:10.1186/s12936-022-04259-7)
Supplement: Supplementary file 1 — Additional file 1: Fig. S1. Scattergrams illustrating (a) malaria-negative sample, (b) P. falciparum positive sample, and (c) inconclusive (MI-RBC abnormal [Abn] scattergram) by XN-31p. SFL: side fluorescence light; FSC: forward scattered light. Blue dots: non-infected RBCs, platelets, and debris; red dots: parasite-infected red blood cells; light blue dots: white blood cells. Fig. S2. Protocol for capillary blood collection. Fig. S3. Flow chart of iRBC count and %iRBC determination by microscopy. Table S1. Performance of XN-31p in comparison to conventional methods with detailed results. Table S2. Fixed and proportional biases observed in the CBCs with 24 hours stored samples [file 12936_2022_4259_MOESM1_ESM.docx]

Supplemental Fig. 1. Scattergrams illustrating (a) malaria-negative sample, (b) *P. falciparum* positive sample, and (c) inconclusive (MI-RBC abnormal [Abn] scattergram) by XN-31p. SFL: side fluorescence light; FSC: forward scattered light. Blue dots: non-infected RBCs, platelets, and debris; red dots: parasite-infected red blood cells; light blue dots: white blood cells.


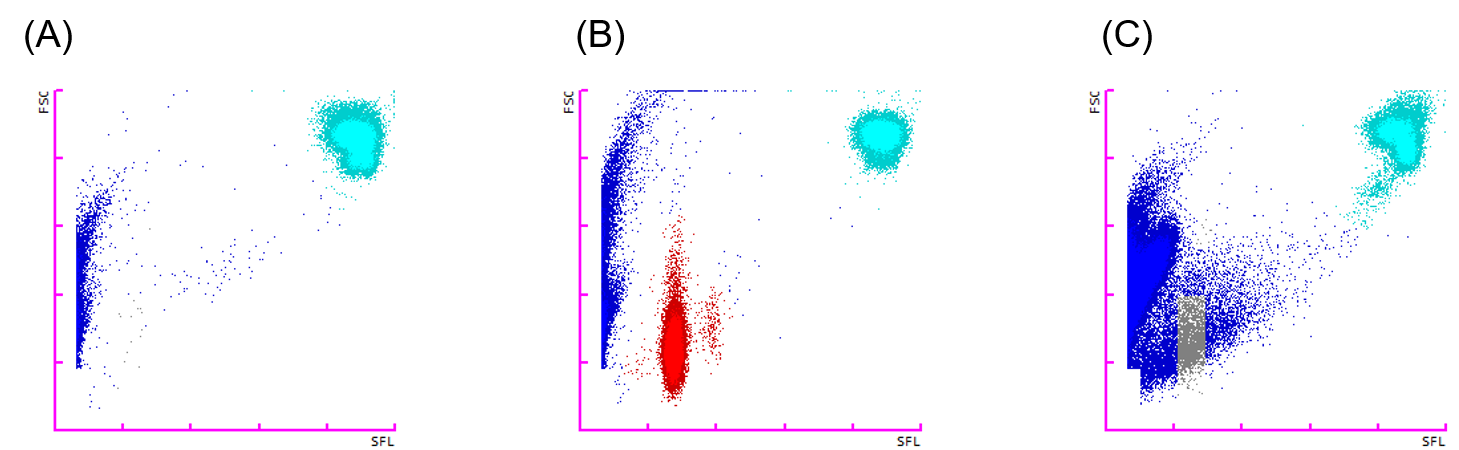


Supplemental Fig. 2. Protocol for capillary blood collection.


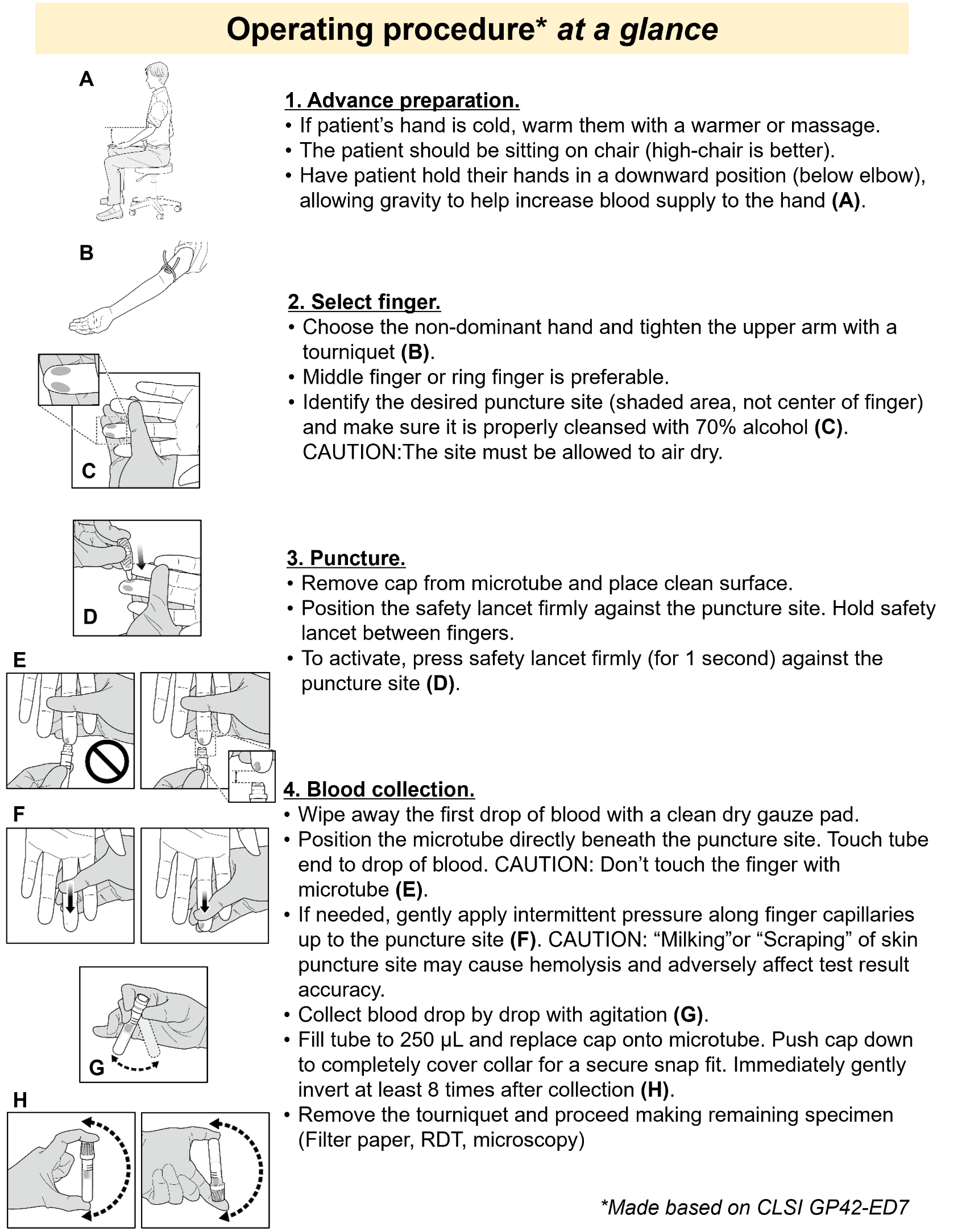


Supplemental Fig. 3. Flow chart of iRBC count and %iRBC determination by microscopy.


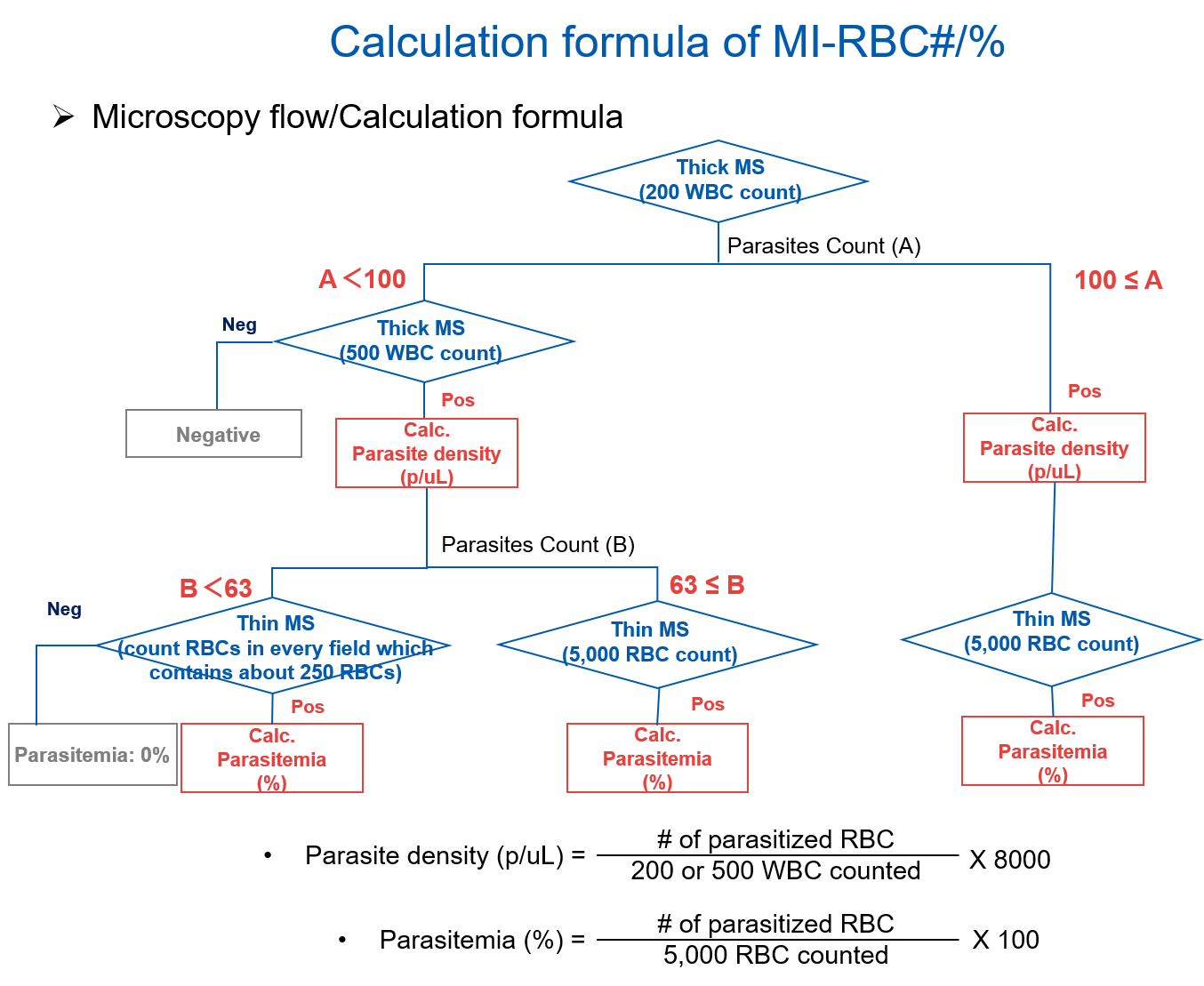


Supplemental Table 1. Performance of XN-31p in comparison to conventional methods with detailed results

|  | | Microscopy | | | RDT | | PCR | | | |
| --- | --- | --- | --- | --- | --- | --- | --- | --- | --- | --- |
|  |  | *P. falciparum* | *P. malariae* | Neg | Pos | Neg | *P. falcipalrum* | *P. ovale* | *P. falciparum* + *P. malariae* | Neg |
| XN-31p  (venous blood) | Malaria?(P.f) | 15 | 1 | 2 | 18 | 0 | 17 | 0 | 1 | 0 |
|  | MI-RBC Abn Scattergram | 0 | 0 | 5 | 0 | 5 | 1 | 0 | 0 | 4 |
|  | Negative | 0 | 0 | 146 | 0 | 146 | 1 | 3 | 0 | 142 |
| XN-31p  (capillary blood) | Malaria?(P.f) | 15 | 1 | 1 | 17 | 0 | 16 | 0 | 1 | 0 |
|  | Malaria?(others) | 0 | 0 | 1 | 1 | 0 | 1 | 0 | 0 | 0 |
|  | MI-RBC Abn Scattergram | 0 | 0 | 11 | 0 | 11 | 1 | 1 | 0 | 9 |
|  | Negative | 0 | 0 | 140 | 0 | 140 | 1 | 2 | 0 | 137 |

“MI-RBC abnormal (Abn) scattergram” is the flag shown in case of inconclusive results.

Supplemental Table 2. Fixed and proportional biases observed in the CBCs with 24 hours stored samples

|  | 24 hours at cool condition (n=47) | | 24 hours at room temperature (n=51) | |
| --- | --- | --- | --- | --- |
|  | Fixed bias | Proportional bias | Fixed bias | Proportional bias |
| WBC | Not detected | Not detected | Yes (p < 0.001) | Yes (rho = 0.382, p < 0.001) |
| RBC | Yes (p < 0.001) | Not detected | Yes (p < 0.001) | Yes (rho = 0.316, p < 0.05) |
| Hb | Yes (p < 0.001) | Not detected | Yes (p < 0.001) | Not detected |
| HCT | Yes (p < 0.001) | Not detected | Yes (p < 0.001) | Not detected |
| Plt | Not detected | Yes (rho = 0.358, p < 0.01) | Not detected | Not detected |

Samples in the room temperature group were kept in an air-conditioned room with temperature set at 22°C, while those in the cool condition were kept in a cooler box with frozen ice packs (2 to 8°C).
